# The importance of feature aggregation in radiomics: a head and neck cancer study

**DOI:** 10.1038/s41598-020-76310-z

**Published:** 2020-11-12

**Authors:** Pierre Fontaine, Oscar Acosta, Joël Castelli, Renaud De Crevoisier, Henning Müller, Adrien Depeursinge

**Affiliations:** 1 Institute of information Systems, University of Applied Sciences Western Switzerland (HES-SO), TechnoArk 3, 3960 Sierre, Switzerland; 2 CLCC Eugene Marquis, INSERM, LTSI - UMR 1099, Univ Rennes, 35000 Rennes, France; 3 Service of Nuclear Medicine and Molecular Imaging, Lausanne University Hospital (CHUV), Lausanne, Switzerland

**Keywords:** Image processing, Machine learning

## Abstract

In standard radiomics studies the features extracted from clinical images are mostly quantified with simple statistics such as the average or variance per Region of Interest (ROI). Such approaches may smooth out any intra-region heterogeneity and thus hide some tumor aggressiveness that may hamper predictions. In this paper we study the importance of feature aggregation within the standard radiomics workflow, which allows to take into account intra-region variations. Feature aggregation methods transform a collection of voxel values from feature response maps (over a ROI) into one or several scalar values that are usable for statistical or machine learning algorithms. This important step has been little investigated within the radiomics workflows, so far. In this paper, we compare several aggregation methods with standard radiomics approaches in order to assess the improvements in prediction capabilities. We evaluate the performance using an aggregation function based on Bags of Visual Words (BoVW), which allows for the preservation of piece-wise homogeneous information within heterogeneous regions and compared with standard methods. The different models are compared on a cohort of 214 head and neck cancer patients coming from 4 medical centers. Radiomics features were extracted from manually delineated tumors in clinical PET-FDG and CT images were analyzed. We compared the performance of standard radiomics models, the volume of the ROI alone and the BoVW model for survival analysis. The average concordance index was estimated with a five fold cross-validation. The performance was significantly better using the BoVW model 0.627 (95% CI: 0.616–0.637) as compared to standard radiomics0.505 (95% CI: 0.499–0.511), mean-var. 0.543 (95% CI: 0.536–0.549), mean0.547 (95% CI: 0.541–0.554), var.0.530 (95% CI: 0.524–0.536) or volume 0.577 (95% CI: 0.571–0.582). We conclude that classical aggregation methods are not optimal in case of heterogeneous tumors. We also showed that the BoVW model is a better alternative to extract consistent features in the presence of lesions composed of heterogeneous tissue.

## Introduction

Radiomics allows quantitative analyses from radiological images with high throughput extraction to obtain prognostic patient information^1^.

Prediction of disease-free survival or the response to the treatment is performed via quantitative image features extracted from diagnostic or pre-treatment images. Previous improvements on radiomics workflows mainly addressed either the features optimization step, i.e. better description the tumor and its environment, or the improvement of machine learning algorithms^2,3^. However, some underlying relations that may exist between radiomics features and outcomes may be hidden due to the way they are quantified in the early stages of the workflow. Region-wise analysis of features is often performed by using low order statistics extracted over the entire region of the lesion. Nevertheless, additional relationships may be revealed by considering intra-regional heterogeneity using specific aggregation functions with feature maps.

The general process and related impact of feature aggregation methods has so far been little investigated in this context. In order to extract collections of scalar measurements that can be used as independent variables for statistical and machine learning algorithms^4^, an aggregation function is required to gather and summarize the operator responses over a considered Region Of Interest (ROI). Classical aggregation functions include first-order measures, which can be computed not only the image itself but also to response maps of image operators such as image filters or co-occurrence matrices. A common established feature aggregation method in radiomics is to compute the average, the variance (e.g. the first four statistical moments) or quantiles (e.g. maximum, minumum) of the distribution of the voxel values inside the ROI.

**Figure 1:**
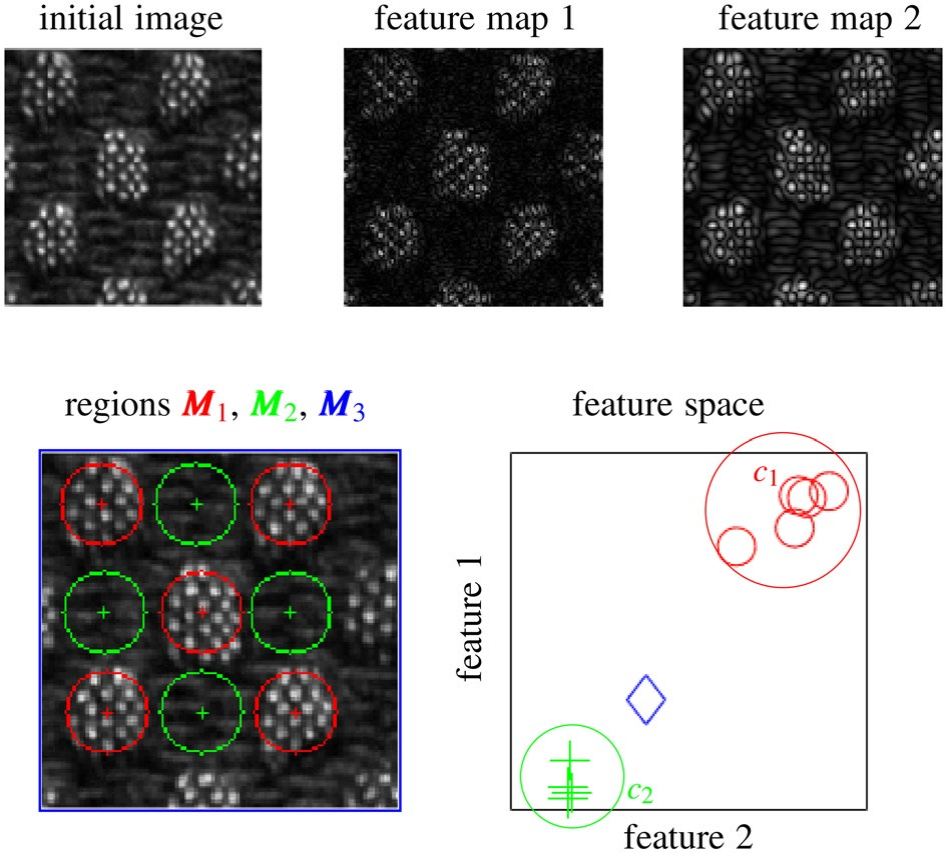
Influence of the size and localization of the ROI ${\varvec{M}}$ for aggregating the feature maps using the average. Each sub-region ${\varvec{M}}_1$ and ${\varvec{M}}_2$ is well separated in the feature space spanned by Simoncelli wavelets and aggregated using the average. The blue region ${\varvec{M}}_3$ (entire image) involves the averaging of non-stationary sub-regions. As a consequence, this blue region does not represent the true content of the image well, because its representation in the feature space (blue diamond) falls in between the true observations (red circles and green crosses). $c_1$ and $c_2$ represent clusters (called *visual words*) found using the BoVW approach allowing to reveal and preserve pattern heterogeneity by relying on an aggregation function that is integrative regarding parts in the feature space.

The average in particular is the most straightforward aggregation function but it is inappropriate when tumors and composing tissue are heterogeneous (i.e. non-stationary). This aspect is illustrated in Fig. 1, where the initial image (fabric) contains two visually distinct sub-regions ${\varvec{M}}_1$ and ${\varvec{M}}_2$, corresponding to the *visual words* $c_1$ and $c_2$, respectively. The sub-regions are also distinct and well-defined in a feature space spanned by the responses of Simoncelli wavelets^5^ and aggregated using the average over the sub-regions ${\varvec{M}}_1$ and ${\varvec{M}}_2$. However, when the feature maps are aggregated over the entire image ${\varvec{M}}_3$, the averaging operation results in an information loss and the resulting scalar features do not correspond to the two distinct patterns observed in the initial image (blue diamond).

In general, integrative aggregation functions such as counting or averaging over ${\varvec{M}}$ are inappropriate for non-stationary feature maps.

Feature aggregation has been extensively studied in computer vision and led to substantial performance improvement in the context of image classification and retrieval. Most notable examples are Bags of Visual Words (BoVW)^6^, Fisher Vectors^7^, and DeepTen^8^. The BoVW is a well-known method in computer vision, more precisely in the field of image classification^9^.

It consists of describing images as a vector of visual words instead of one single scalar, where each visual word is a relatively homogeneous (stationary) region revealed via clustering (e.g. $c_1$ and $c_2$ in Fig. 1). Fisher Vectors extend the BoVW framework by adding second-order moments of the features. DeepTen was introduced in the context of Convolutional Neural Networks (CNN). It is an encoding network which can be inserted between the convolutional layers and the final layer. This encoding layer learns an inherent dictionary and also affects the weights in the convolutional part during the training step.

Surprisingly, feature aggregation was little investigated in the context of radiomics. Three studies focused on the importance of feature aggregation in the context of lung cancer. Cirujeda et al.^10^ proposed an aggregation method based on feature covariances on top of a Riesz-wavelet decomposition, which outperformed feature aggregation based on the average. Cherezov et al.^11^ used clustering of a circular harmonic wavelet coefficients and showed superior categorization of cancer aggressiveness when compared to classical radiomics features. And Hou et al.^12^ evaluated the performance of Bag-of-features-based radiomics for differentiating ocular adnexal lymphoma and idiopathic orbital inflammation from contrast enhanced MRI. In this paper, we investigate the importance of the feature aggregation step. To this end, we compare several standard approaches (count, average, variance) to the BoVW method applied to various feature types including filters and gray-level matrices (co-occurrences, run-length). The comparison is performed in the context of overall survival analysis with a multicentric cohort of head and neck cancer and PET-FDG and CT scans from 214 patients. Radiomics models were already proposed for head and neck cancer^13,14^, but no study focused on the impact of the aggregation function on the model performance.

This paper is organized as follows. Patient characteristics are detailed in “Patient data” section. Section “Image operators, feature maps and aggregation functions” lays the distinct fundamental elements of feature extraction by introducing image operators, their response maps (also called feature maps) and aggregation functions. The latter are further defined in the particular case of image filters and gray-level matrices (i.e. co-occurrences and run-length). Specifically considered features and their parameters are described in “Feature extraction” section. The fundamentals of the BoVW method and its specific use on with radiomics image operators are described in “Bags of visual words” section. The validation method used to estimate the performance of the proposed radiomics models for overall survival analysis is detailed in “Model validation” section. Corresponding results, interpretation and general conclusions are provided in Sections “Results” and “Discussions and conclusion”, respectively.

## Material and methods

### Patient data

214 patients from four centers (Rennes, Lausanne, Besançon and Lorient) were retrospectively analyzed. The patients were aged between 18 and 75 years with an average age of 62, stage III or IV (AJCC 7th edition) with no surgery before RT, nor history of cancer, nor metastasis at diagnosis and a minimal follow-up of 3 months. All patients were treated with ChemoRadioTherapy (CRT) or RadioTherapy (RT) combined with Cetuximab. The outcome studied is dead (positive) or alive (negative) in a context of overall survival analysis. The study was approved by the institutional ethical committees (NCT02469922 and Commission cantonale d’éthique de la recherche sur l’être humain: CER-VD 2018-01513). Patient details are listed in Table 1.

**Table 1:** Patient characteristics.

Cohort	# patient	Mean age, years (SD)	Stage (AJCC)		# events
Rennes	103	62 (9)	III	22	63
			IV	81	
Besançon	34	63 (8)	III	13	16
			IV	21	
Lorient	16	NC	III	5	5
			IV	11	
Lausanne	61	63 (9)	III	20	7
			IV	41	

### PET/CT image acquisition

All patients underwent FDG PET/CT for staging at most 8 weeks before RT. For three centers, an injection of 4 Mbq/kg of 18F-FDG was given to the patient who fasted at least four hours. After a 60 minutes uptake period of rest, images were taken using the Discovery ST PET/CT imaging system (GE Healthcare) or the Siemens Biograph 6 True Point PET/CT scanner (Siemens Medical Solutions). First, CT (120 kV, 80 mA, 0.8 s rotation time, slice thickness 3.75 mm) was performed, followed by the PET immediately afterwards. A similar protocol was used for the last center; however, a smaller injection of 3.5 Mbq/kg of 18F-FDG was used with the Discovery D690 TOF PET/CT (GE Healthcare). For each patient, Gross Tumor Volume-Tumor (GTV-T) were manually segmented on each PET/CT images by the same radiation oncologist. A ROI was computed by adding a 3D margin of 5 mm to GTV-T. More details can be found in Castelli *et al*.^15^.

### Image operators, feature maps and aggregation functions

In this section, we use the general theoretic framework for radiomic analysis introduced in^16^ to define and isolate the role and responsibilities of the aggregation step. We consider discrete images $I[{\varvec{k}}]$ indexed by the vector ${\varvec{k}}=(k_1,k_2,k_3)\in {\mathbb {Z}}^3$. In general terms, a radiomics image analysis approach can be characterized by a set of *N* local operators ${\mathscr {G}}_{n}$ and their corresponding spatial supports ${\varvec{G}}_{n} \subset {\mathbb {Z}}^{3}$. The expression ${\mathscr {G}}_{n}\{f\}[{\varvec{k}}_0] \in {\mathbb {R}}$ represents the application of the operator ${\mathscr {G}}_{n}$ to the image *I* at location ${\varvec{k}}_0$ and provide a scalar-valued response. The operator ${\mathscr {G}}_{n}$ is applied at every location ${\varvec{k}}\in {\mathbb {Z}}^{3}$ in the image by systematically sliding its corresponding support ${\varvec{G}}_n$ over the entire image (For the sake of simplicity, we consider that the support of the image *I* is ${\mathbb {Z}}^3$). This process yields response maps $h_{n}[{\varvec{k}}]$ (also called feature maps) as $h_{n}[{\varvec{k}}] = {\mathscr {G}}_{n}\{I\}[{\varvec{k}}]$. Finally, $h_{n}[{\varvec{k}}]$ can be summarized over a ROI ${\varvec{M}}$ to compute, via an aggregation function such as the average or maximum, a scalar feature $\eta _n$.

#### Filters

This first type of image operators considered belongs to a group of approaches called convolutional and are based on topological operators called filters. The image operator ${\mathscr {G}}$ is fully characterized by a topological function $g[{\varvec{k}}]$, where ${\mathscr {G}}$ is linear and its application to the image *I* at the position ${\varvec{k}}_0$ is obtained via the scalar product of *I* and *g* as

1 $$\begin{aligned} {\mathscr {G}}\{I\}[{\varvec{k}}_0] = \langle I[\cdot ],g[{\varvec{k}}_0 - \cdot ]\rangle . \end{aligned}$$

The full feature map is obtained via the convolution as $h[{\varvec{k}}]=(g*I) [{\varvec{k}}]$. One classical way to aggregate the feature map $h[{\varvec{k}}]$ to obtain a scalar valued feature $\eta$ is to compute the average as $\eta = \frac{1}{|{\varvec{M}}|} \sum _{{\varvec{k}}\in {\varvec{M}}} h[{\varvec{k}}]$ which is integrative and where $|{\varvec{M}}|$ denotes the number of elements (i.e. voxels) in the region ${\varvec{M}}$. It is worth noting that the absolute value or the energy of the feature map must be computed for filters $g[{\varvec{k}}]$ that are zero-mean.

#### Gray-level matrices

Gray-level matrices are based on binary operators detecting the presence or absence of a given configuration of gray-levels starting at the location ${\varvec{k}}_0$. These configurations can include co-occurrences^17^, run-lengths^18^ or even size zones^19^. The first two are detailed below.

Gray-Level Co-occurrence Matrices (GLCM) GLCMs^17^ are based on a quantized image $I_{\Lambda }[{\varvec{k}}]\in (1,\dots ,\Lambda )$ with $\Lambda$ the number of gray-levels (e.g. 8, 16, 32) and binary operators ${\mathscr {G}}$ that are detecting co-occurences between two gray levels $(\lambda _i,\lambda _j)$ observed at the position pairs ${\varvec{k}}_0$ and ${\varvec{k}}_0 + \Delta {\varvec{k}}$. As such, GLCMs are based on a collection of operators defined as

2 $$\begin{aligned} {\mathscr {G}}_{\lambda _{i},\lambda _{j},\Delta {\varvec{k}}}\{f_{\Lambda }\}[{\varvec{k}}_0] = {\left\{ \begin{array}{ll} 1 &{} \hbox {if }f_{\Lambda }[{\varvec{k}}_0] = \lambda _i\hbox { and }f_{\Lambda }[{\varvec{k}}_0 + \Delta {\varvec{k}}] = \lambda _{j}, \\ 0 &{} \hbox {otherwise}. \end{array}\right. } \end{aligned}$$

This collection of responses is aggregated in an integrative fashion over ${\varvec{M}}$ by constructing a co-occurrence matrix, which simply counts the responses of the various operators and organizes them in a square co-occurrence matrix $\mathrm {C}$ of dimension $\Lambda ^{2}$ indexed by ($\lambda _{i},\lambda _{j}$). Then, a collection of scalar texture measurements $\varvec{\eta }$ is obtained by computing quantities (e.g., cluster prominence, correlation, entropy, also called *Haralick features*) from $\mathrm {C}$.

Gray-Level Run-Length Matrices (GLRLM) Within a quantized image $I_{\Lambda }[{\varvec{k}}]$, GLRLMs^18^ operators detect strides of contiguous aligned voxels with identical gray-level value $\lambda$, length $||\Delta {\varvec{k}}||$ and direction $\Delta {\varvec{k}}$ as

3 $$\begin{aligned} {\mathscr {G}}_{\lambda ,\Delta {\varvec{k}}}\{I_\Lambda \}({\varvec{k}}_0) = {\left\{ \begin{array}{ll} 1 &{} \hbox {if a stride of gray-level }\lambda \hbox { starting at the position }{\varvec{k}}_0\hbox { and ending at } {\varvec{k}}_0+\Delta {\varvec{k}}\hbox { is detected,} \\ 0 &{} \hbox {otherwise}. \end{array}\right. } \end{aligned}$$

The aggregation is similar to GLCMs that count the response of the operators and organizes them in a run length matrix $\mathrm {R}$ of dimension $\Lambda \times \Delta$ indexed by ($\lambda ,||\Delta {\varvec{k}}||$), where $\Delta$ is the number of lengths $||\Delta {\varvec{k}}||$ considered. Collections of scalars $\varvec{\eta }$ are computed from these matrices (e.g., short run emphasis, grey level non-uniformity, run percentage).

### Feature extraction

**Figure 2:**
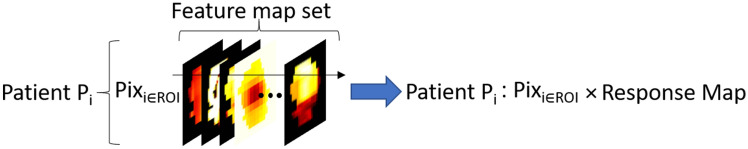
For each patient $P_i$, the 42 feature maps are concatenated into a matrix where each coefficient voxel of the ROI is a 42-dimensional vector.

Before the feature extraction step, we convert CT images into Hounsfield Units (HU) and PET images into Standardized Uptake Value (SUV). We resampled images (isotropic resampling to 1mm cubic voxels) to allow adequate image scale comparisons of all texture features across image series. For step (i), from those resampled images, we extract 42 features (21 on CT and 21 on PET) and their response map from each of the 214 patients, using our own software tools that were benchmarked with the reference values provided by the Image Biomarker Standardisation Initiative (IBSI^20^). A list of these features is provided in Table 2. We focused on those where aggregation is critical, i.e. filters and gray-level texture matrices (also called second-order). Shape features were excluded since they do not require an aggregation step. Classical separable Wavelets (e.g. Haar, Daubechies) were not included as they are generating many irrelevant directional feature maps (e.g. XXX, XXY, XYZ, etc...), which is discussed in Section 4.6 of Depeursinge, et al.^21^. This is illustrated in 2D in Fig. 2. For each patient $P_i$ we compute a collection of feature maps $h[{\varvec{k}}]$. Every pixel belonging to the ROI is considered as an observation in a feature space spanned by the 42 feature maps. It is worth noting that the creation of feature maps is uncommon for gray-level texture matrices. Then, we compute the gray-level matrices and related quantitative features over $5 \times 5\times 5$ cubic sliding windows for GLCMs and GLRLMs. In this window, we defined a collection of space directions. In 3D, the number of possible spatial directions is 13 for $\Delta {\varvec{k}} = 1\,\hbox {mm}$ displacements. We also chose $\Delta {\varvec{k}} = 2 \,\hbox {mm}$ with the same 13 directions. This resulted in a total of 26 distinct offsets and we calculated 26 corresponding GLCMs. We computed the value of the quantitative features for every voxel position $\varvec{k_0}$ to generate the response maps before using an aggregation function (e.g. average) over the ROI to compute scalar-valued features. The same 13 directions, radius and aggregation methodology was used for GLRLM features. This size of the sliding window was chosen as a trade-off between locality of the features (limiting the influence of surrounding objects) and the ability of the features to capture texture patterns with larger size^22^.

**Table 2:** The list of the detailed features used in the study.

Family	Feature	Quantitative feature
Filter-based	Laplacian of Gaussian Gabor Sobel	Sigma $= 2\hbox {mm}$, radius $= 4\hbox {mm}$ Sigma $= 11/3$, freq. $= 0.4$, radius $= 4 \,\hbox {mm}$ Kernel size = 3 × 3 × 3
Grey-level texture matrices	GLRLM Radius $= 2 \,\hbox {mm}$ Angles $=$ Half of all directions (3D), symmetrical Discretization $= 64$ grey levels	ShortRunEmphasis LongRunEmphasis GreyLevelNonuniformity RunLengthNonuniformity LowGreyLevelRunEmphasis HighGreyLevelRunEmphasis ShortRunLowGreyLevelEmphasis ShortRunHighGreylevelEmphasis LongRunLowGreyLevelEmphasis LongRunHighGreyLevelEmphasis
	GLCM Radius $= 2 \,\hbox {mm}$ Angles $=$ Half of all directions (3D), symmetrical Discretization $= 64$ grey levels	Energy InverseDifferenceMoment Entropy HaralickCorrelation ClusterShade ClusterProminence Inertia Correlation

### Bags of visual words

The Bag of Visual Words (BoVW) model is an image extension of the bag of words model used in the field of information retrieval and text analysis^6,23^. Building a BoVW model is performed in three steps: (i) compute feature maps, (ii) reveal dictionaries of visual words using clustering and (iii) compute frequency histograms by counting occurrences of each visual word to describe an entire ROI.

Then, step (ii) relies on the clustering (e.g. *k*-means, Gaussian mixtures, DBSCAN^24^) of the feature space created in step (i). Each cluster center is considered as a visual word and the set of clusters constitute the visual dictionary of our set of training images. This process is illustrated in Fig. 1 where the two clusters (i.e. visual words) $c_1$ and $c_2$ correspond to the two distinct texture patterns present in the initial image. We chose the Gaussian mixture model as clustering algorithm in order to define clusters based on both mean and variance. The most interesting particularity of the BoVW method is that step (ii) acts as a feature aggregation function that is integrative by parts in the feature space, which allows revealing and preserving distinct homogeneous sub-regions.

Step (iii) uses the results of steps (i) and (ii) to assign each voxel of the ROI to a cluster $c_i$, thus populating a histogram of visual words of dimension *k* that can be further used as a collection of scalars $\varvec{\eta }$ for machine learning models (see “Model validation” section).

### Model validation

This section details the workflow used to evaluate the radiomics model’s performance using the head and neck cohort described in “Patient data” section, and in particular to test our hypothesis that feature aggregation has an important role in radiomics. To estimate the influence of the feature aggregation method on the survival prediction performance, we pooled the image data from the four centers and randomly divided it five times into a training cohort and a validation cohort using a stratified shuffling method. We used a Cox–Lasso regression model^25^ to predict a Hazard Score (HS) and further computed Harrell’s C-index^26^ as our performance measure to estimate the quality of survival analysis. We created the dictionary based on each training fold. The BoVW model is compared to four other baseline models based on classical aggregation methods, as well as one univariate model based on the volume of the ROI (i.e. tumor) only^27^, which can be seen as the most basic aggregation function based on the count of the number of voxels inside the ROI. To summarize, we evaluate the following six models:

1. *Classical radiomics* This model uses the classical aggregation functions described in “Image operators, feature maps and aggregation functions” section, i.e. the average for filters and the count followed by the collection of scalars for the gray-level texture matrices. Sliding-window-based feature maps are therefore not used in this case.
2. *Average-variance* Average and variance inside the ROI based on the (sliding-window) feature maps computed as described in step (i) of “Feature extraction” section.
3. *Average* Average only inside the ROI from the feature maps,
4. *Variance* Variance only inside the ROI from the feature maps.
5. *Volume* Univariate model based on the volume of the ROI only.
6. *BoVW* The BoVW model as described in “Bags of visual words” section.

For all methods, the final feature collections $\varvec{\eta }$ were standardized to *z*-scores using the mean and standard deviation estimated on the training folds.

**Figure 3:**
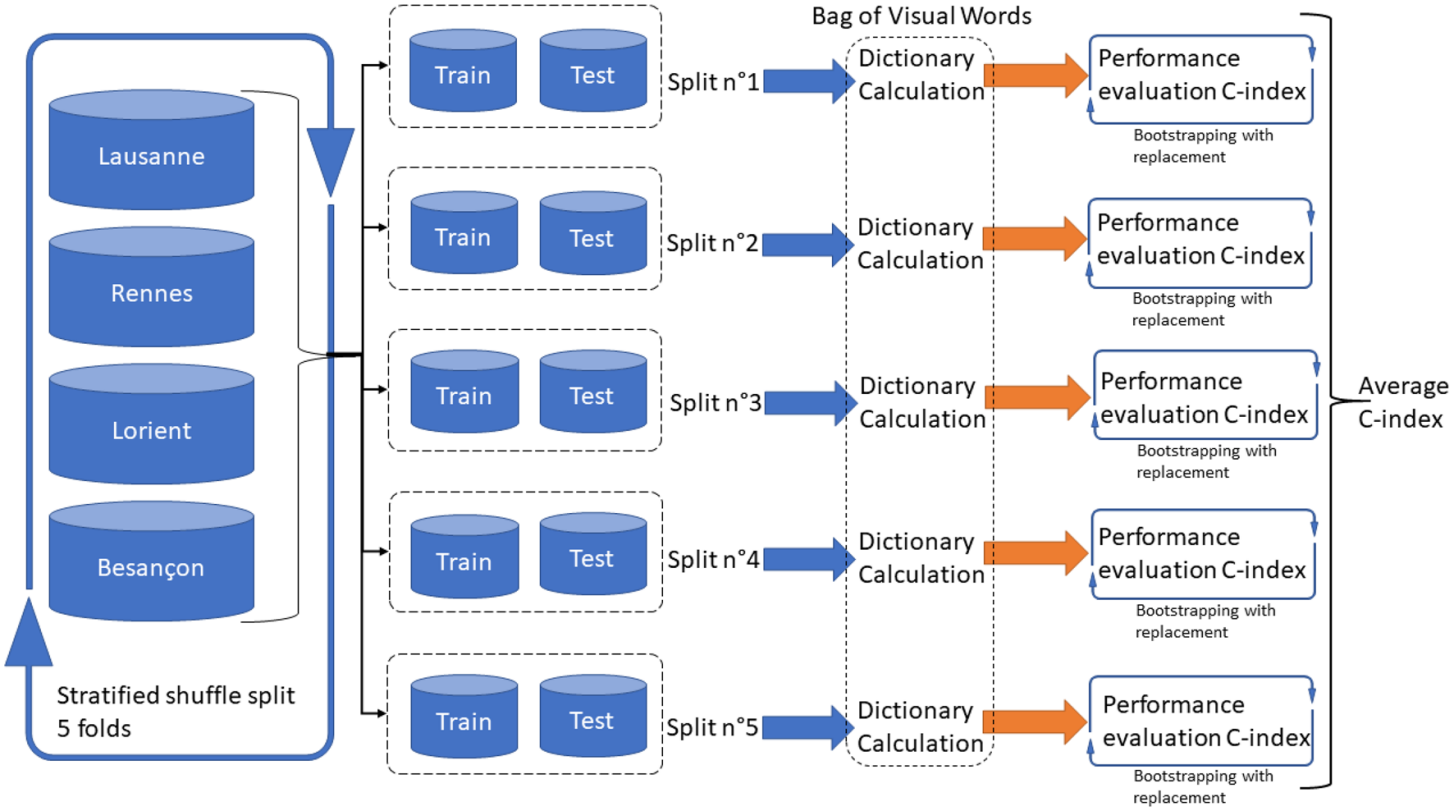
Proposed validation strategy using the multi-centric cohort of head and neck cancer.

In each fold, we evaluate the six models together by bootstrapping with replacement (1000 times) and calculating the C-index. The five folds yields 5000 estimations of the C-index for each model, which we summarize with averages and their Confidence Intervals (CI) at 95%. This validation strategy is shown and summarized in Fig. 3.

### Ethical approval

All procedures performed in studies involving human participants were in accordance with the ethical standards of the institutional and/or national research committee and with the 1964 Helsinki Declaration and its later amendments or comparable ethical standards. The study was approved by the institutional ethical committees (NCT02469922 and Commission cantonale d’éthique de la recherche sur l’être humain: CER-VD 2018-01513).

### Informed consent

Informed consent was obtained from all individual participants included in the study.

## Results

**Figure 4:**
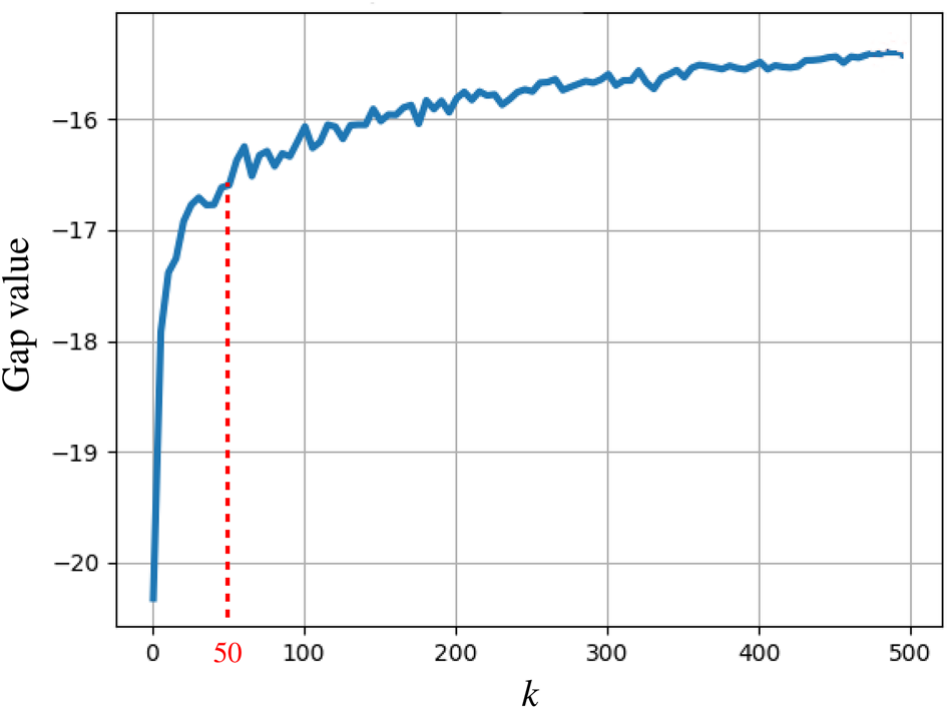
The number of clusters *k* is chosen based on the Gap value (higher is better) computed on the entire dataset. We chose $k=50$ clusters (i.e. visual words) as a very large number of cluster is required to significantly increases the Gap value beyond $k=50$.

**Figure 5:**
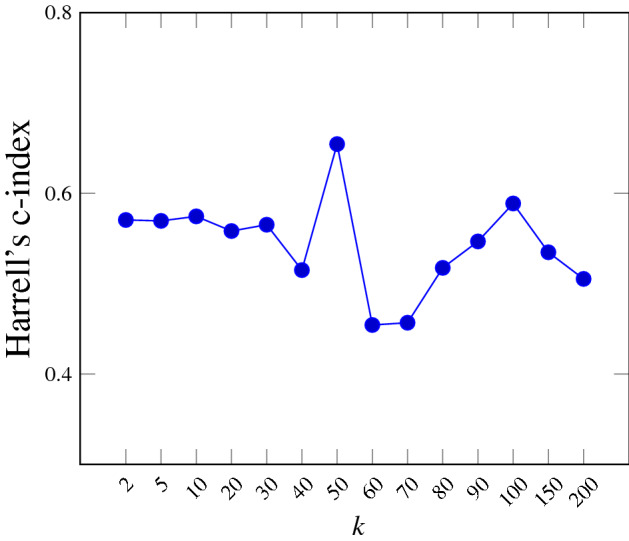
Influence of *k* on the performance of the survival model measured using the C-index.

We first investigate the influence of the number of clusters *k* (i.e. the number of visual words) on the performance of the BoVW model. Several methods exist to determine the optimal *k*, including the Elbow^28^, Silhouette^29^ or Gap statistic^30^. In this study, we use the Gap statistic method as it is based on the measure of intra-cluster variation. Using the entire dataset, Fig. 4 reveals that $k=50$ constitutes an interesting trade-off between the number of words and the ability to capture data heterogeneity. Using the validation scheme described in “Model validation” section, the influence of *k* on the performance of the BoVW model is shown in Fig. 5. Based on these results, we fixed $k=50$ for the remaining experiments, which is also close to the dimensionality of the initial number of features extracted (i.e. 42).

**Figure 6:**
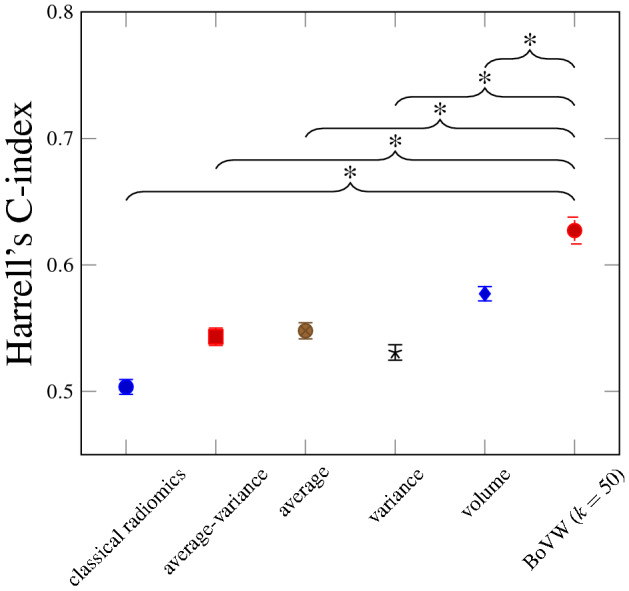
Average C-indices and 95% CIs for the six proposed models based on various feature aggregation methods. *$p<0.01$.

**Figure 7:**
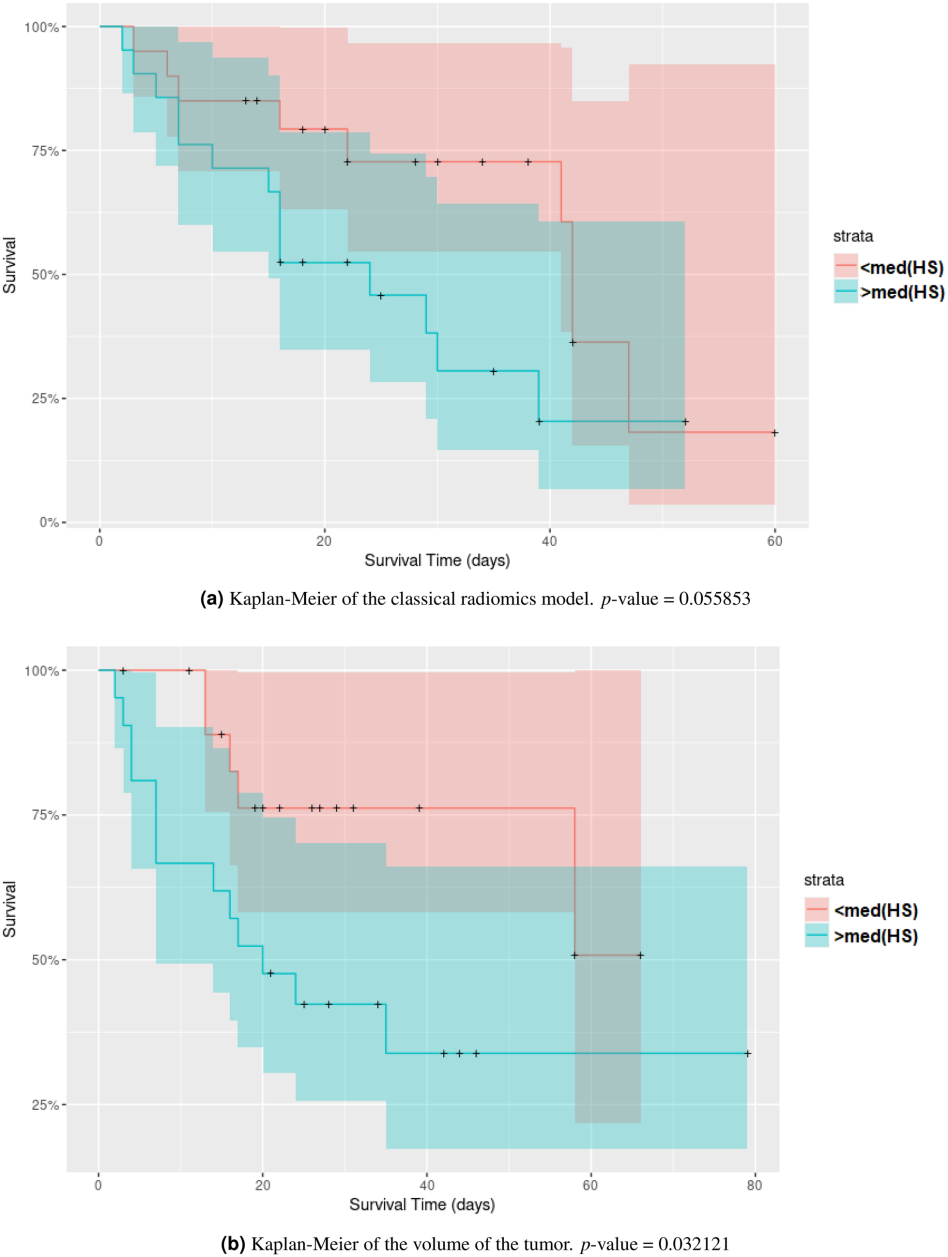

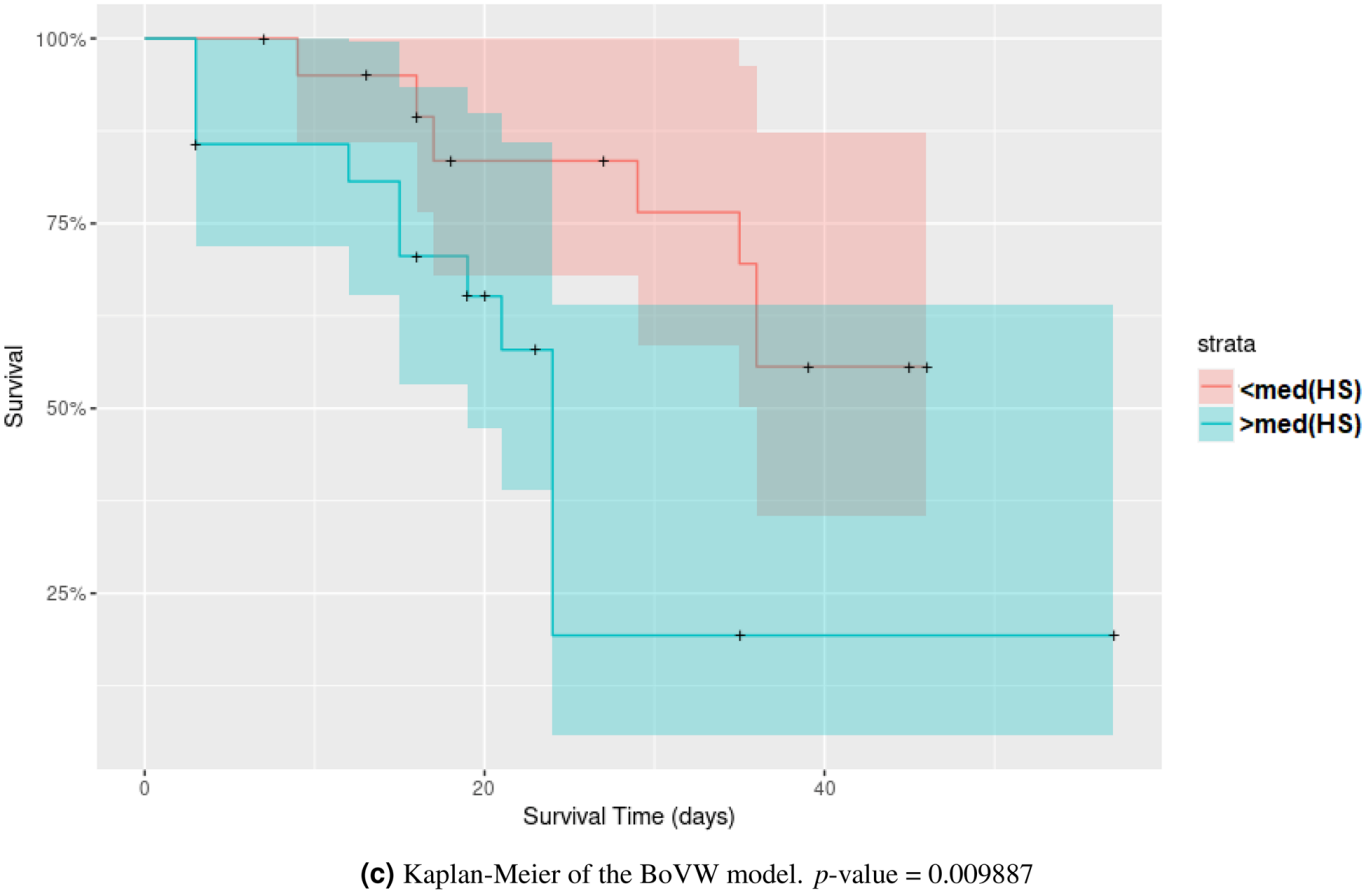
Kaplan–Meier curves using a risk stratification into two groups as defined by the median value of the HS (“Model validation” section).

Figure 6 compares the C-index values for all six models presented in “Model validation” section using the validation method explained in Fig. 3. Table 3 lists the average C-index values for each method. The BoVW approach allows improving the performance in a statistically significant way when compared to all other aggregation methods as well as the volume. The classical radiomics model does not deliver predictions that are significantly better than random. We derived the Kaplan–Meier curve for three models (Fig. 7): Classical radiomics (Fig. 7a), Volume (Fig. 7b) and BoVW (Fig. 7c). The group stratification is based on the median of the HS provided by the prediction of the unseen test set for one train/test split: the split with a performance that was the closest to the respective observed average C-index (see Table 3) was used. The Kaplan–Meier curves (Fig. 7c) of the BoVW model suggests that the latter allows to separate the patients with distinct survival characteristics better than the other approaches.

**Table 3:** Harrell’s C-indices for the six proposed models.

	Mean (lower bound-upper bound) (95% CI)
Classical radiomics	0.505 (0.499–0.511)
Average–variance	0.543 (0.536–0.549)
Average	0.547 (0.541–0.554)
Variance	0.530 (0.524–0.536)
Volume	0.577 (0.571–0.582)
BoVW	0.627 (0.616–0.637)

## Discussions and conclusion

Radiomics is becoming increasingly important in particular in oncology. It allows to non-invasively predict response to treatment or to characterize tumor type and aggressiveness. The main assumption of the work described in this article is that heterogeneous tumors require more advanced feature aggregation methods than the classical integrative or quantile-based methods that are commonly used in radiomics.

Averaging or using the maximum voxel value in non-stationary response maps entails the risk of mixing or discarding different sources of information.

As observed in Fig. 6 and Table 3, the method used to aggregate information inside the ROIs can significantly impact the performance of the model in overall survival analysis for head and neck cancer. The BoVW method to aggregate feature maps allowed to improve the performance of survival models with statistical significance. This result can be attributed to the fact that the BoVW relies on the integration of parts for feature aggregation, allowing to reveal and preserve sub-regions in non-stationary feature maps. Figure 6 shows that no classical feature aggregation method could outperform a simple model relying on the tumor volume solely.This can be partly explained by the large heterogeneity of our dataset with four clinical centers with different scanner manufacturers. This generates variations in radiomics features but has a limited impact on the measure of the volume. The Kaplan–Meier analysis (Fig. 7) showed that both BoVW and volume models (Fig. 7b,c) have significant prognostic performance (i.e. *p* value = 0.009 and *p* value = 0.032, respectively), where the BoVW model allowed best stratification. By contrast, the classical radiomics model (Fig. 7a) is not significant with a *p* value = 0.055 (which is consistent with the observed average C-index of this model). This demonstrating the possibility of specific risk assessment in head and neck cancer, which is consistent with reported results of previous studies^13–15^.

This work constitutes a proof-of-concept demonstrating the importance of feature aggregation in radiomics studies. We recognize several limitations. First, as we focused on the feature aggregation step, the feature extraction step was not specifically optimized for the task at hand and simply relies on a classical radiomics feature set. Second, the histogram of visual words used in the BoVW is very sparse since it relies on hard cluster assignments. Therefore, the Cox–Lasso model might struggle to work with such sparse data matrices, which we plan to further investigate in future work.
